# Elucidation of Molecular Identity of the *W3* Locus and Its Implication in Determination of Flower Colors in Soybean

**DOI:** 10.1371/journal.pone.0142643

**Published:** 2015-11-10

**Authors:** Gyu Tae Park, Jagadeesh Sundaramoorthy, Jeong-Dong Lee, Jeong Hoe Kim, Hak Soo Seo, Jong Tae Song

**Affiliations:** 1 School of Applied Biosciences, Kyungpook National University, Daegu, Korea; 2 Department of Biology, Kyungpook National University, Daegu, Korea; 3 Department of Plant Bioscience, Seoul National University, Seoul, Korea; McGill University, CANADA

## Abstract

The wide range of flower colors in soybean is controlled by six independent loci (*W1*, *W2*, *W3*, *W4*, *Wm*, and *Wp*). Among these loci, mutations in the *W3* locus under the *w4* allelic background (i.e., *w3w4*) produce near-white flowers, while the *W3w4* genotype produces purple throat flowers. Although a gene encoding dihydroflavonol 4-reductase, *DFR1*, has been known to be closely associated with the *W3* locus, its molecular identity has not yet been characterized. In the present study, we aimed to determine whether *DFR1* is responsible for allelic variations in the *W3* locus. On the basis of the sequence of a *DFR* probe, *Glyma*.*14G072700* was identified as a candidate gene for *DFR1*, and nucleotide sequences of *Glyma*.*14G072700* from cultivars with previously validated genotypes for the *W3* locus were determined. As a result, a number of nucleotide polymorphisms, mainly single-base substitutions, between both coding and 5′-upstream region sequences of the *W3* and *w3* alleles were identified. Among them, an *indel* of 311-bp in the 5′-upstream region was noteworthy, since the *Glyma*.*14G072700* in all the *w3* alleles examined contained the *indel*, whereas that in all the *W3* alleles did not; the former was barely expressed, but the latter was well expressed. These results suggest that *Glyma*.*14G072700* is likely to correspond to *DFR1* for the *W3* locus and that its expression patterns may lead to allelic color phenotypes of *W3* and *w3* alleles under the *w4* allelic background.

## Introduction

Soybean [*Glycine max* (L.) Merr.] exhibits a wide range of flower colors, such as dark purple, purple, light purple, pink, magenta, near-white, and white. Sorting analysis of variations in flower color helps in understanding the evolutionary history of soybean cultivars [1]. Variations in flower colors in soybean are mainly ascribed to six different genetic loci (*W1*, *W2*, *W3*, *W4*, *Wm*, and *Wp*) [2]. Among them, *W1*, *Wm*, and *Wp* encode flavonoid 3′5′-hydroxylase, flavonol synthase, and flavanone 3-hydroxylase, respectively; *W2* corresponds to a MYB transcription factor that regulates the pH of vacuolar sap [3–11].

In soybean, anthocyanin pigments play a major role in flower color. In the anthocyanin biosynthesis pathway, dihydroflavonol 4-reductase (DFR) acts as an essential enzyme that catalyzes the production of leucoanthocyanidins, which are, at ensuing steps, converted to anthocyanins. The relationship between purple and white colors with *DFR* was studied in many ornamental and horticultural plants. *DFR* exhibited a low level of *MYB* and *DFR* transcripts in white rather than purple *Phalaenopsis* petals and sepals [12]. Similarly, in the orchid *Dendrobium sonia*, white tissues of petals rather than purple tissues showed repressed *DFR* expression [13]. In *Nicotiana tabacum*, the wild type flowers of which have pink petals, a white-flower mutant was characterized as a DFR-deficient one [14]. In soybean, genes that encode DFR enzymes cosegregate with two loci, namely, *W3* and *W4*, and they act epistatic to each other under the *W1* allelic background [15,16]. Soybean accessions with *W3W4* produce dark purple flowers; *w3W4*, purple; *W3w4*, purple throat; and *w3w4*, near-white [17,18], indicating that allelic variations in the *W3* locus under the *w4* recessive allelic background are clearly distinguished by the color phenotypes (i.e., purple throat and near-white flowers).

As for other color phenotypes, low levels of *DFR2* expression or aberrant transcripts of *DFR2* were found to be associated with mutations in the *W4* locus under the *w3* recessive allelic background, leading to different shades of purple flowers, such as dilute purple, pale purple, and light purple [16,18,19].

To isolate the *DFR* gene responsible for anthocyanin biosynthesis in soybean, Wang et al. [20] developed a *DFR* probe, a 200-bp fragment from a genomic PCR clone (pDFR200) that contained the partial sequence of a *DFR* gene. Using the *DFR* probe, Fasoula et al. [15] performed restriction fragment length polymorphism (RFLP) analysis and revealed that the *W3* locus was cosegregated with a *DFR* probe. However, these studies have not culminated in determining the molecular identity of the *W3* locus. Herein, we tried to determine whether *DFR1* is responsible for allelic variations in the *W3* locus.

## Results

### Molecular identification of *DFR1*

Using RFLP analysis with the restriction enzyme *Hae*III, Fasoula et al. [15] identified a DNA fragment of ~1.2-kb that covered the *DFR* probe in lines harboring the *W3* allele (L70-4422) and found that the fragment was replaced by a longer one (i.e., ~1.7-kb) in the *w3* alleles (Clark 63, L68-1774, L72-2181, and L69-4776). The study also revealed the presence of an invariant fragment of ~1.3-kb in all the lines examined, indicating that the ~1.3-kb fragment is independent of the *W3* locus.

**Fig 1:**
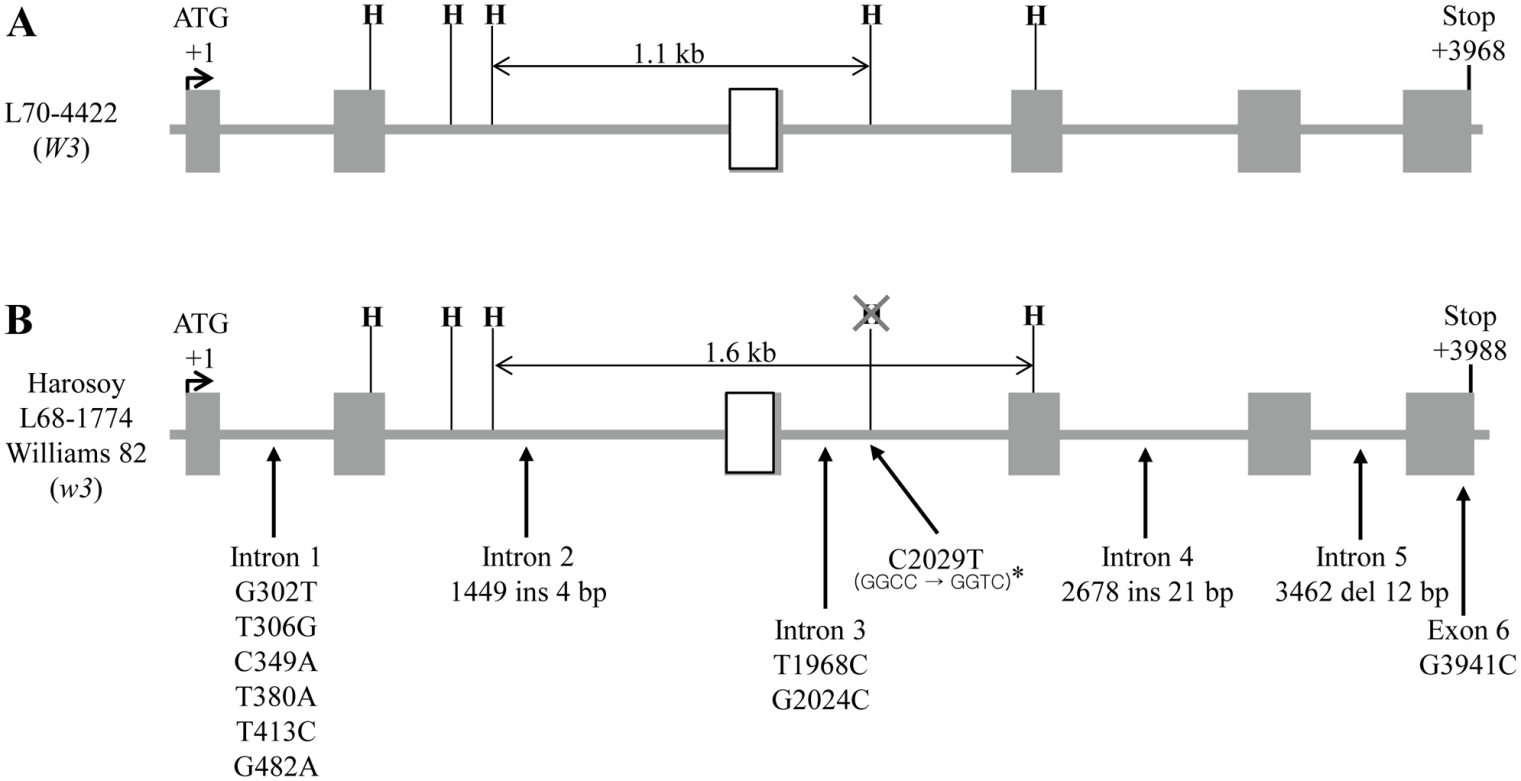
*DFR1* gene structure and polymorphism between *W3* and *w3* alleles. (A) The *W3* allele (GenBank accession number for *DFR1* of L70-4422; KT721361) shows five *Hae*III restriction sites and a 1.1-kb restriction fragment covering the *DFR* probe. Boxes and solid lines represent exons and introns, respectively. The white box indicates the *DFR* probe region. H denotes *Hae*III restriction sites (GGCC). (B) The *w3* allele (GenBank accession numbers for *DFR1* of Harosoy and L68-1774; KT721359 and KT721360, respectively) shows several differences when compared with the *W3* allele: a number of single-base substitutions, a deletion, and three insertions in introns as well as a single-base substitution in exon 6. Note that no polymorphism was detected between three cultivars with the *w3* allele. The asterisk indicates deletion of the *Hae*III restriction site caused by a single-base substitution (T to C) in intron 3. Ins and del denote insertion and deletion.

To determine whether *DFR1* is responsible for allelic variations in the *W3* locus, we first searched for candidate genes in soybean genome database (Phytozome version 10.3; http://phytozome.jgi.doe.gov/pz/portal.html) with nucleotide sequences that are highly similar to that of the *DFR* probe, the sequence of which was obtained from Wang et al. [20]. As a result, we found that *Glyma*.*14G072700*, actually its exon 3, showed 100% identity with the *DFR* probe sequence, indicating that it is the most probable candidate for *DFR1* (Table 1). *Glyma*.*14G072700* was annotated in the database as a gene that encodes a bifunctional DFR/flavanone 4-reductase (FNR). In addition, four genes were selected as possible candidates, since they showed >75% similarities to the *DFR* probe (Table 1). We then compared the *Hae*III restriction fragment patterns of Fasoula et al. [15] with those of *Glyma*.*14G072700* from *W3* (L70-4422) and *w3* alleles (Harosoy, L68-1774, and Williams 82). Sequence analysis revealed that *Glyma*.*14G072700* from the *W3* allele showed five restriction sites for *Hae*III and that the length of the restriction fragment covering the *DFR* probe region was 1,097-bp (Fig 1A); all the *w3* alleles showed only four restriction sites, since one was lost because of a single-base substitution (C to T) at position 2,029 in intron 3 (Fig 1B). Elimination of the *Hae*III restriction site lengthened the restriction fragment covering the *DFR* probe to 1,594-bp. In brief, the restriction fragment lengths of *Glyma*.*14G072700* from *W3* and *w3* alleles were ~1.1- and ~1.6-kb, respectively, which corresponded to the ~1.2- and ~1.7-kb fragments obtained by Fasoula et al. [15]. The result suggests that *Glyma*.*14G072700* may be the *DFR1* gene responsible for allelic variations in the *W3* locus.

**Table 1:** List of genes selected as candidates for the *W3* locus.

Gene	Gene annotation	Identity with *DFR* probe	Size by *Hae*III (bp)*
*Glyma*.*14G072700*	Bifunctional DFR/FNR (DFR1)	100.0%	1,594
*Glyma*.*17G252300 ~ Glyma*.*17G252400*	Not annotated	91.8%	1,237
*Glyma*.*02G158700*	Bifunctional DFR/FNR	85.1%	4,261
*Glyma*.*17G252200*	Bifunctional DFR/FNR (DFR2)	79.5%	479 & 552
*Glyma*.*14G072800*	Bifunctional DFR/FNR	75.9%	1,867 & 2,598

* *Hae*III restriction fragment length covering the *DFR* probe.

On the other hand, a gene positioned between *Glyma*.*17G252300* and *Glyma*.*17G252400*, although not annotated in the database, had 92% similarity to the *DFR* probe, and produced a 1,237-bp-long restriction fragment (Table 1). Interestingly, the length is similar to that of the invariant fragment (~1.3-kb) described by Fasoula et al. [15]. Besides, three other candidate genes (*Glyma*.*02G158700*, *Glyma*.*17G252200*, and *Glyma*.*14G072800*) exhibited *Hae*III restriction fragments whose lengths were widely different from those of *Glyma*.*14G072700* as well as those described by Fasoula et al. [15]. The results altogether indicate that *Glyma*.*14G072700* indeed corresponds to *DFR1*, which was previously proposed to be closely associated with the *W3* locus.

### Analysis of allelic variations in the coding region of *DFR1*

**Fig 2:**
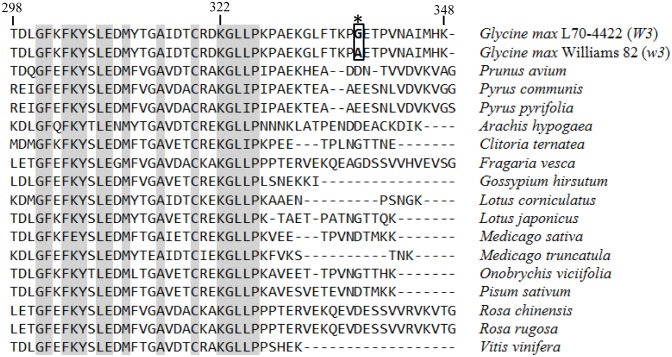
Amino acid sequence alignment of DFR proteins. Identical amino acid residues are highlighted in grey. The asterisk indicates the position of a single amino acid residue substitution between *W3* and *w3* alleles. GenBank accession numbers of amino acid sequences of DFRs are as follows: *Prunus avium*, AHL45016; *Pyrus communis*, AAO39818; *Pyrus pyrifolia*, AFF60412; *Arachis hypogaea*, AEX07281; *Clitoria ternatea*, BAF49294; *Fragaria vesca*, XP_004291858; *Gossypium hirsutum*, ACV72642; *Lotus corniculatus*, AAV71171; *Lotus japonicus*, AFK35141; *Medicago sativa*, AEI59122; *Medicago truncatula*, XP_003638261; *Onobrychis viciifolia*, AEF14420; *Pisum sativum*, AII26023; *Rosa chinensis*, AHF58604; *Rosa rugosa*, AIU34714; and *Vitis vinifera*, NP_001268144.

Next, we analyzed nucleotide sequences of the coding regions of *DFR1* genes from both alleles (*W3*: L70-4422; *w3*: L68-1774, Harosoy, and Williams 82). Compared with *DFR1* from the *W3* allele, introns of the *w3* allele had several single-base substitutions, one 12-bp deletion, and two insertions (Fig 1). In addition, the last exon of the *w3* allele had a single-base substitution (G to C) at position 3,941, consequently substituting Ala for Gly at position 338 of the amino acid sequence of the DFR1 protein (Figs 1 and 2).

To infer whether the single-base substitution in the last exon could lead to changes in DFR1 protein activity, multiple alignment of amino acid sequences of DFR1-related proteins from 17 different plant species was constructed. The alignment showed that the C-terminal regions of DFR1 homologues were divergent between different species (Fig 2). Although whole amino acid sequences of *W3* and *w3* alleles of *G*. *max* are identical, except for the substitution at the last exon, C-termini of DFR1 homologues from other plant species are variable in length and composition of amino acid residues. It is, therefore, conceivable that the substitution in the last exon may not cause an alteration in DFR1 protein activity and, thus, may not be the cause for allelic variations in the *W3* locus.

### Expression patterns of *DFR1* in *W3* and *w3* alleles

**Fig 3:**
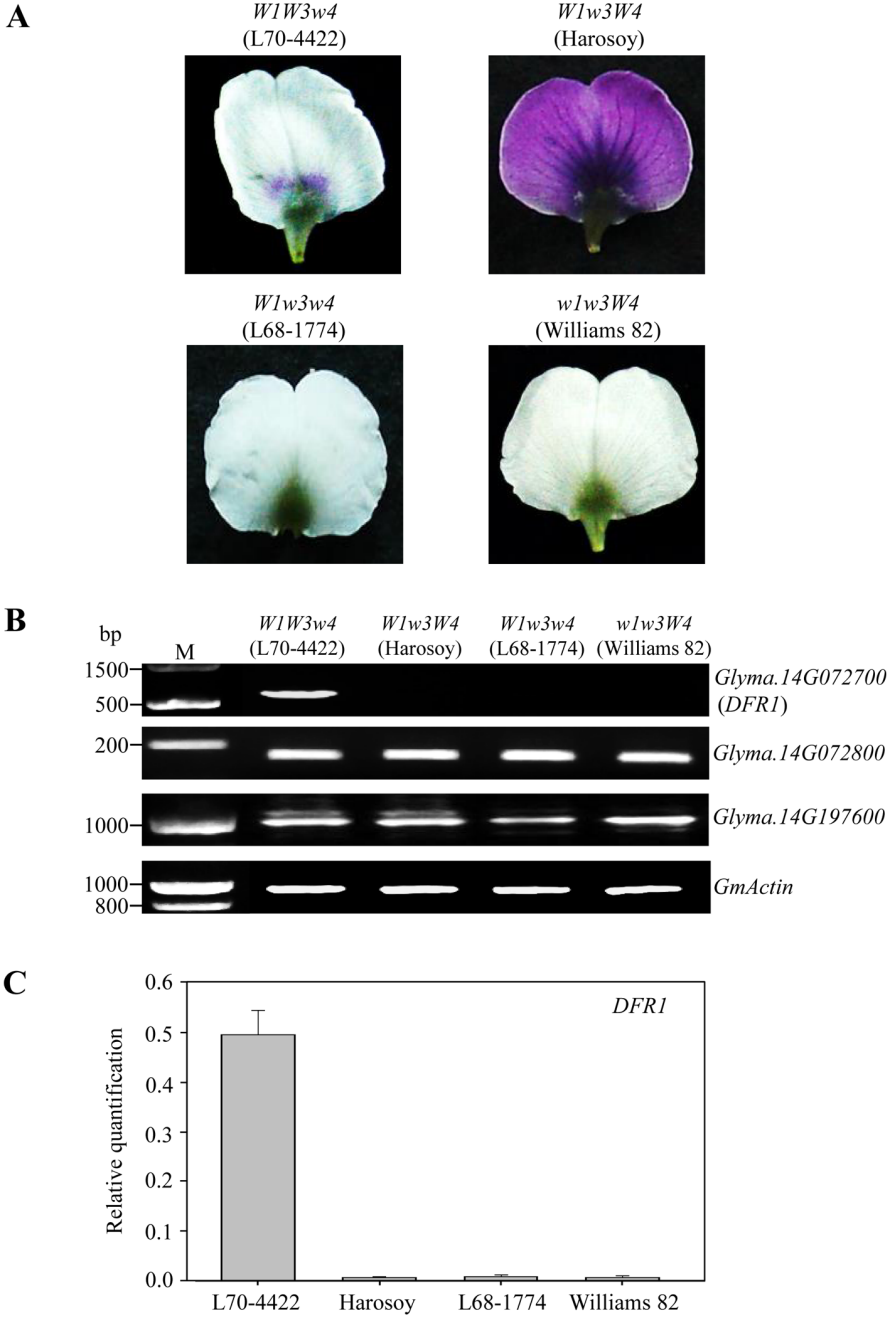
Gene expression profiles of *W3* and *w3* alleles from soybean cultivars. (A) Photographic images of soybean genotypes *W1W1W3W3w4w4* (L70-4422) with purple throat flowers, *W1W1w3w3W4W4* (Harosoy) with purple flowers, *W1W1w3w3w4w4* (L68-1774) with near-white flowers, and *w1w1w3w3W4W4* (Williams 82) with white flowers. (B) RT-PCR analysis of *Glyma*.*14G072700* (*DFR1*) and its related genes (*Glyma*.*14G072800* and *Glyma*.*14G197600*) of the indicated lines. *GmActin* expression was used as the loading control. (C) qRT-PCR analysis of *Glyma*.*14G072700* (*DFR1*). Values are means and standard deviations from three biological replicates. *DFR1* expression was normalized with *constitutive gene* 7 (*Cons7*) as the reference gene.

We performed reverse transcription–polymerase chain reaction (semi-quantitative and quantitative RT-PCR) analyses to determine the expression level of *DFR1* in standard petals of *W3* and *w3* plants (Fig 3). In both analyses, *DFR1* expression was high in the *W3* allele with purple throat flowers, whereas it was barely detected in the *w3* allele with near-white flowers (Fig 3B and 3C). We also analyzed expression levels of two other genes, *Glyma*.*14G072800*, which is located at the position subsequent to *DFR1* and annotated as a *DFR*, and *Glyma*.*14G197600*, which is positioned at the same chromosome as that with *DFR1* and has 52.3% amino acid sequence similarity to DFR1. As a result, both genes (*Glyma*.*14G072800* and *Glyma*.*14G197600*) were expressed independently of the allelic variations of the *W3* locus (Fig 3B). The results indicate that the expression patterns of *DFR1* are tightly correlated with purple throat and near-white flowers.

### Analysis of the *DFR1* 5′-upstream region

**Fig 4:**
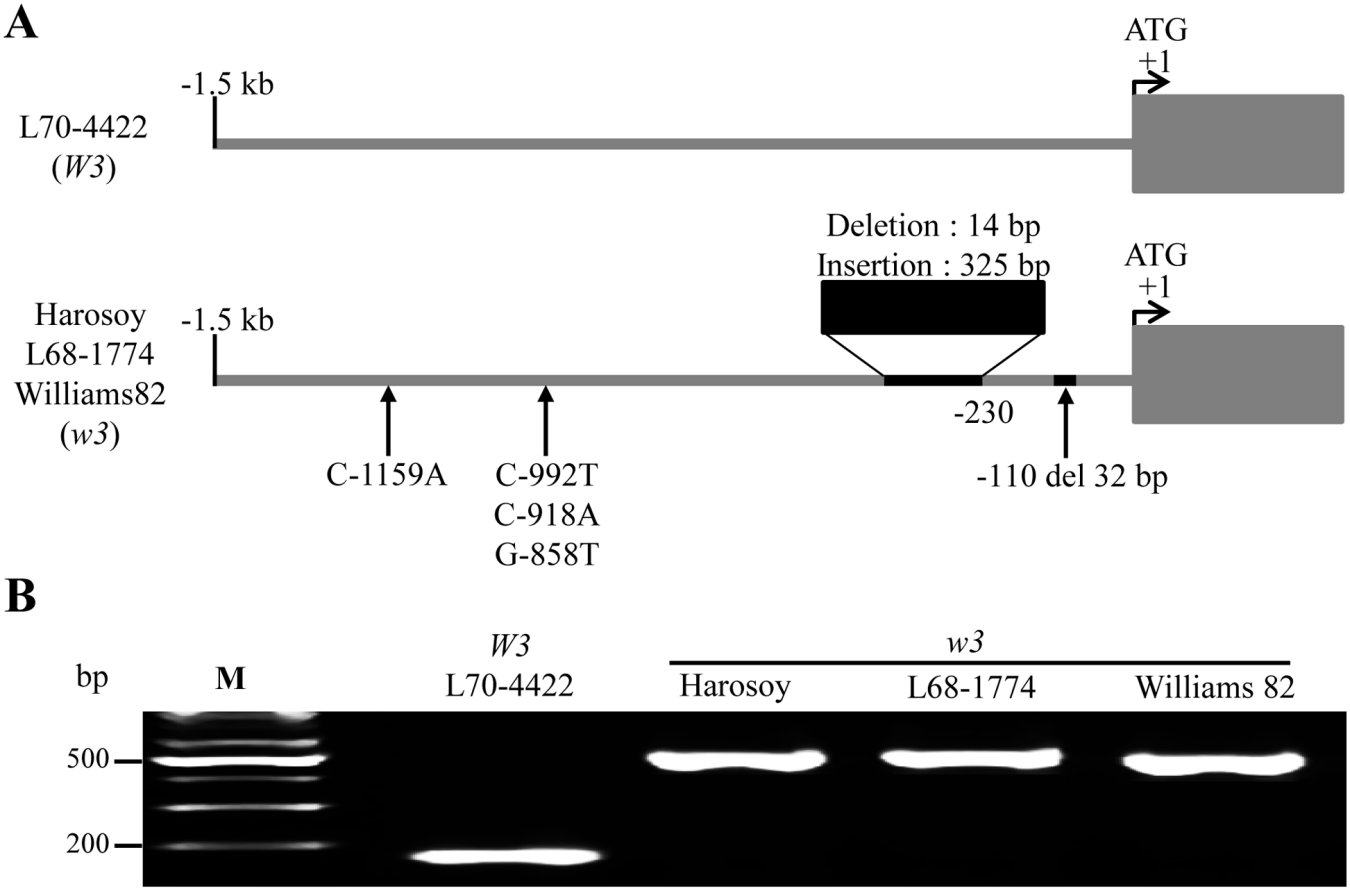
Sequence analysis of the 5′-upstream region of *DFR1*. (A) Diagrams of 5′-upstream sequences up to -1.5-kb of *DFR1* (upper: *W3*, lower: *w3*). Note that no polymorphism was detected between three cultivars with the *w3* allele. (B) A correlation between the *indel* and expression of *DFR1*. The shorter (~200-bp) and longer fragments (~500-bp) result from *DFR1* of *W3* and *w3* alleles, respectively.

We observed a significant difference in the expression levels of *DFR1* between *W3* and *w3* alleles. Herein, we compared the nucleotide sequences of the 5′-upstream region (up to -1.5-kb from the start codon) of *DFR1* from both alleles to search for variations that could be responsible for *DFR1* differential expression. Compared with the *DFR1* 5′-upstream region of the *W3* allele, that of the *w3* alleles contains four single-base substitutions, a 32-bp deletion, and an *indel* of 311-bp (325-bp insertion and 14-bp deletion) at position -230 (Fig 4A). PCR using primers flanking the *indel* amplified a DNA fragment of ~200-bp in the *W3* allele and a longer one (~500-bp) in all the *w3* alleles because of the presence of the *indel* (Fig 4B). The results are consistent with the *DFR1* expression patterns and, thus, with the color phenotypes observed in *W3* and *w3* alleles.

**Fig 5:**
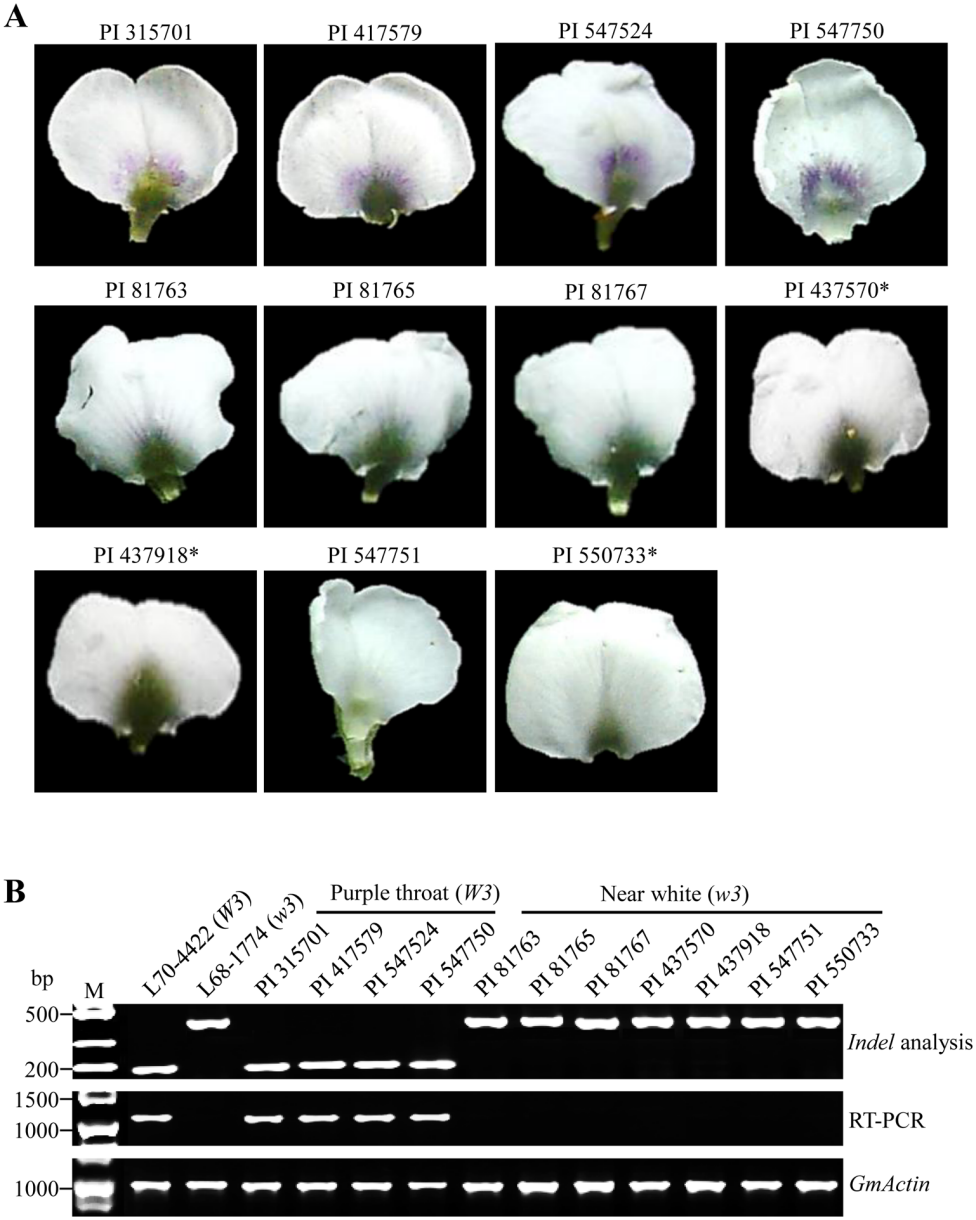
*Indel* and expression analysis of *DFR1* in 11 soybean accessions. (A) Photographs of standard petals of purple throat (upper row) and near-white accessions (middle and bottom rows). Asterisks indicate the accessions that were described as purple throat flowers in the USDA-GRIN database. (B) Determination of genotypes of purple throat and near-white phenotypes with the *indel* marker in the 5′-upstream region of *DFR1*. *GmActin* expression was used as the loading control.

To confirm the correlation between the *indel* and *DFR1* expression, we extended the analysis to more soybean accessions (four purple throats with *W3* and seven near-whites with *w3* from United States Department of Agriculture–Germplasm Resource Information Network database (USDA-GRIN; http://www.ars-grin.gov/). We selected those accessions, as they harbor *W3* or *w3* alleles under the *w4* allelic background (Fig 5A). Otherwise, it would be difficult to phenotypically distinguish between *W3* and *w3* because under the *W4* allelic background, *W3* and *w3* alleles produce purple and dark purple flowers, respectively.

As a result, all purple throat accessions showed ~200-bp amplification products in the *indel* analysis and were accompanied by normal expression of *DFR1*; this was in accordance with the results for the *W3* allele (L70-4422) (Fig 5B). In contrast, all near-white accessions showed ~500-bp products and barely expressed for *DFR1*, which was consistent with the results of the *w3* allele (L68-1774). It should be noted that three accessions (i.e., PI 437570, PI 437918, and PI 550733) were described as purple throat accessions in the USDA-GRIN database. However, those accessions developed near-white flowers under our field conditions (see Materials and Methods), and *indel* and expression analyses indicated that those three accessions actually fitted better into the category of the *w3* allele. These results indicate that the *indel* of the 5′-upstream region is tightly correlated with the expression level of *DFR1*.

## Discussion

We demonstrated that *Glyma*.*14G072700* corresponds to *DFR1* and thus the *W3* locus and that there are many nucleotide polymorphisms in its introns, exon 6, and 5′-upstream region. Among them, the *indel* of the 5′-upstream region is noteworthy for not only distinguishing *W3* from *w3* but also characterizing their expression patterns and resulting in different shades of flower colors. Therefore, it is a tempting speculation that the *indel* of *DFR1* may be a cause for allelic variations in the *W3* locus.

In plants, *cis*-elements control phenotypes by regulating gene expression so that their mutations or modifications can lead to dramatic changes in tissue-specific expression patterns [21,22,23]. A previous study showed that modifications in *cis*-regulatory elements of *DFR* genes caused limited expression and thus resulted in aberrant anthocyanin synthesis in Caryophyllales [24]. The nucleotide polymorphisms exhibited by the *DFR1* 5′-upstream region, notably the 311-bp *indel*, may compromise *DFR1* expression due to interference of the binding of certain transcription factors to their target sites.

Structural genes involved in the anthocyanin biosynthesis are tightly regulated by transcription factors [25]. In petals of *Phalaenopsis amabilis*, for instance, absence of the expression of an MYB transcription factor gene was responsible for the absence of *DFR* expression and lack of anthocyanin pigments [12]. On the basis of the *cis*-element finding tool (www.dna.affrc.go.jp/PLACE), we analyzed the *indel* sequence. The *indel* sequence harbors putative *cis*-elements, such as ARR1AT (NGATT), CACTFTPPCA1 (YACT), and CAAT box (CAAT). ARR1AT is a cytokinin response regulator that acts as transcriptional activator in Arabidopsis and rice [26]. YACT is a tetranucleotide motif responsible for mesophyll-specific gene expression in C_4_ plants [27]. CAAT box is important for the tissue-specific promoter activity of *LegA* in pea [28]. Thus, the 311-bp *indel* of *w3* allele may inhibit the expression of *DFR1* by binding of certain transcription factors to the *cis*-element. However, we cannot rule out that the 14-bp deletion or other polymorphisms may also influence *DFR1* expression.

Interestingly, coding and 5′-upstream region sequences of all three *w3* accessions (Harosoy, L68-1774, and Williams 82) analyzed in this study showed no difference in sequences, which makes us envisage the possibility that the origin of *w3* alleles may be the same. However, we need to check whether the other seven *w3* alleles tested also have the same sequences, which polymorphism critically affects the expression of *DFR1*, and how the polymorphism affects its expression.

## Materials and Methods

### Plant material

The following soybean cultivars were analyzed: L70-4422 with purple throat flowers (*W1W1W3W3w4w4*), L68-1774 with near-white flowers (*W1W1w3w3w4w4*), Harosoy with purple flowers (*W1W1w3w3W4W4*), and Williams 82 with white flowers (*w1w1w3w3W4W4*) [29]. In addition, PI accessions from USDA-GRIN (four purple throat and seven near-white flower accessions) were used for the PCR experiments (Table 2). All soybean accessions used in this study were grown in the experimental fields of Kyungpook National University (Gunwi, 36°07′ N, 128°38′ E, Republic of Korea).

**Table 2:** List of soybean (*G*. *max*) accessions utilized in genomic and expression analyses of *DFR1*.

Phenotype	Accession
Purple throat	PI 547524 (L70-4422)
	PI 315701
	PI 417579
	PI 547524
	PI 547750
Near-white	PI 547498 (L68-1774)
	PI 81763
	PI 81765
	PI 81767
	PI 437570 *
	PI 437918 *
	PI 547751
	PI 550733 *
Purple	PI 548573 (Harosoy)
White	PI 518671 (Williams 82)

*Accessions were described as purple throat flowers in observations in the USDA-GRIN soybean database. However, we observed their petals to be near-white.

### Isolation of DNA and nucleotide sequence analysis

Genomic DNA of the soybean accessions was isolated from trifoliolate leaves by using the CTAB method [30]. To amplify the exon, intron, and 5′-upstream region of *DFR1*, PCR was performed using the following profile: initial denaturation at 94°C for 5 min, 40 cycles of denaturation at 94°C for 20 s, annealing at 58°C for 40 s, and extension at 72°C for 1 min; a final extension was performed at 72°C for 5 min. PCR products were separated using 1.2% agarose gel, stained with ethidium bromide and visualized under UV light, and finally subjected to sequencing (Solgent, Korea). The primers used for the amplification and sequencing of *DFR1* are listed in Table 3.

**Table 3:** List of primers used in this study.

Gene	Forward primers (5′ to 3′)	Reverse primers (5′ to 3′)
**RT-PCR**		
*Glyma*.*14G072700*	AAATGGGTTCAGCATCCGAAA	AGCAAGTTGCACAGCCATCA
*Glyma*.*14G072800*	CTCAAGAGAGAAGCTTTGATG	TCAGATTTTGGCCTTCACGC
*Glyma*.*14G197600*	CAACAGCAACAGGGTGACA	GCAGCAGCATCATTTAAGAAAGG
*GmActin*	GTTTGCGACAATGGAACAGGAATGGTTAAG	TAATCTTCATGCTACTTGGGGC
**qRT-PCR**		
*Glyma*.*14G072700*	GTTGTCGGTCCCTTTCTGAT	TACCTCCCTTCCACTTCTGG
*Cons7*	ATGAATGACGGTTCCCATGTA	GGCATTAAGGCAGCTCACTCT
***Indel* analysis**		
*Glyma*.*14G072700*	CCCCTTAAAAACTGCTCCCATT	GGAGACCAAAGAATTACTAGTGAGTGA

### Isolation of RNA and cDNA synthesis

Total RNA was isolated from freeze-dried standard petals of soybean accessions by using the phenol-chloroform and lithium chloride precipitation methods [31]. RNA samples were treated with DNaseI to remove contaminating DNA (TaKaRa, Japan). First-strand cDNA was synthesized by reverse transcription of total RNA with an oligo-dT_(20)_ primer and Superscript III, according to the manufacturer’s instructions (Invitrogen, Carlsbad, CA, USA).

### Semi-quantitative RT-PCR analysis

To determine the transcript level of *DFR1* (*Glyma*.*14G072700*), PCR was performed using the first-strand cDNA. Two more candidate genes (*Glyma*.*14G072800* and *Glyma*.*14G197600*) and a housekeeping gene (*GmActin*) were also analyzed, and the primers used are listed in Table 3.

### qRT-PCR analysis

qRT-PCR was performed using the LightCycler^®^ 480 Real-Time PCR System (Roche, Germany). qRT-PCR (20 μl) required 2 μl of first-strand cDNA, 10 pmol of forward and reverse primers, and 10 μl of SYBR green I Master (Roche, Germany). The soybean gene *Cons7* was used as the reference gene [32]. Experiments were performed in triplicate. The following PCR cycle was used: 95°C for 5 min, followed by 45 cycles at 95°C for 10 s, 58°C for 10 s, and 72°C for 20 s. qRT-PCR data and PCR efficiencies were analyzed using the LightCycler^®^ 480 software (Roche, Germany). The primers used in this analysis are listed in Table 3.

### *Indel* analysis

To distinguish between *W3* and *w3* alleles, genetic markers were developed on the basis of the *indel* found in the 5′-upstream region. PCR was performed using the following profile: initial denaturation at 94°C for 5 min, followed by 40 cycles of denaturation at 94°C for 30 s, annealing at 58°C for 30 s, and extension at 72°C for 1 min and a final extension at 72°C for 5 min. The upstream and downstream primers used are listed in Table 3. PCR products for the *w3* allele were longer than those for the *W3* allele because of the existence of the 311-bp *indel* (325-bp insertion and 14-bp deletion) in the 5′-upstream region.
